# A Low-Sodium Diet Boosts Ang (1–7) Production and NO-cGMP Bioavailability to Reduce Edema and Enhance Survival in Experimental Heart Failure

**DOI:** 10.3390/ijms22084035

**Published:** 2021-04-14

**Authors:** Ranjana Tripathi, Ryan D. Sullivan, Tai-Hwang M. Fan, Radhika M. Mehta, Inna P. Gladysheva, Guy L. Reed

**Affiliations:** 1 Department of Medicine, University of Arizona College of Medicine–Phoenix, Phoenix, AZ 85004, USA; ranjanatripathi2006@gmail.com (R.T.); ryansullivan@arizona.edu (R.D.S.); radhikammehta@gmail.com (R.M.M.); 2 Department of Medicine, University of Tennessee Health Science Center, Memphis, TN 38163, USA; tfan1@uthsc.edu

**Keywords:** dietary sodium restriction, dilated cardiomyopathy, edema, nitric oxide, angiotensin (1–7), ACE-2

## Abstract

Sodium restriction is often recommended in heart failure (HF) to block symptomatic edema, despite limited evidence for benefit. However, a low-sodium diet (LSD) activates the classical renin-angiotensin-aldosterone system (RAAS), which may adversely affect HF progression and mortality in patients with dilated cardiomyopathy (DCM). We performed a randomized, blinded pre-clinical trial to compare the effects of a normal (human-equivalent) sodium diet and a LSD on HF progression in a normotensive model of DCM in mice that has translational relevance to human HF. The LSD reduced HF progression by suppressing the development of pleural effusions (*p* < 0.01), blocking pathological increases in systemic extracellular water (*p* < 0.001) and prolonging median survival (15%, *p* < 0.01). The LSD activated the classical RAAS by increasing plasma renin activity, angiotensin II and aldosterone levels. However, the LSD also significantly up-elevated the counter-regulatory RAAS by boosting plasma angiotensin converting enzyme 2 (ACE2) and angiotensin (1–7) levels, promoting nitric oxide bioavailability and stimulating 3′-5′-cyclic guanosine monophosphate (cGMP) production. Plasma HF biomarkers associated with poor outcomes, such as B-type natriuretic peptide and neprilysin were decreased by a LSD. Cardiac systolic function, blood pressure and renal function were not affected. Although a LSD activates the classical RAAS system, we conclude that the LSD delayed HF progression and mortality in experimental DCM, in part through protective stimulation of the counter-regulatory RAAS to increase plasma ACE2 and angiotensin (1–7) levels, nitric oxide bioavailability and cGMP production.

## 1. Introduction

Dilated cardiomyopathy (DCM) is a major cause of heart failure (HF) with reduced ejection fraction (rEF), which is often an irreversible condition in the absence of heart transplantation or mechanical circulatory support [1,2]. Symptomatic HFrEF is characterized by progressive decline in left ventricular function and heart dilation together with pathological sodium and extracellular fluid retention (edema) in the lungs and/or peripheral tissues and cavities, which may cause breathlessness, fatigue, progressive decline in the quality of life, disability and death [1,2,3,4]. Since edema is caused by accumulation of sodium and water, sodium restriction is often recommended in HF to block symptomatic edema [1,5,6,7,8]. The American College of Cardiology Foundation (ACCF) and American Heart Association (AHA) recommend sodium intake be reduced to ≤1500 mg/day for the general population and for most patients with DCM, prior to edema development (stages A–B HF); these guidelines also support sodium restriction for the patients with stages C–D HF, but do not specify a level [1,5,8,9]. The European Society of Cardiology guidelines for HF states that it is common to restrict sodium intake to <2000 mg/day, yet does not give a recommendation with an evidence level [10]. At present, the impact of low-sodium intake on HF progression and death is controversial and the underlying mechanisms are unclear [1,6,7,10,11,12,13].

Sodium and fluid homeostasis is complex. Sodium retention, extracellular fluid volume, renal function and blood pressure are affected by interdependent actions of the renin-angiotensin-aldosterone system (RAAS), natriuretic peptide (NP) system and nitric oxide (NO)-mediated pathways [3,4,5,14,15,16,17]. Under physiological conditions, dietary sodium restriction activates the intra-renal and systemic classical RAAS, increasing active renin production and upregulating angiotensin II and aldosterone, in order to stimulate renal sodium reabsorption and maintain body fluid volume [3,4,15]. The NP system, NO-mediated pathways and the counter-regulatory angiotensin converting enzyme 2 (ACE2)-angiotensin (1–7) (Ang (1–7)) axis of the RAAS counterbalances activation of the classical RAAS. They promote diuresis, natriuresis, and vasodilation by enhancing NO bioavailability and by initiating 3′-5′-cyclic guanosine monophosphate (cGMP)-related signaling [14,16,17,18,19,20,21,22].

In HFrEF, under normal sodium intake, classical RAAS activation may compensate initially for impaired cardiac function, yet its prolonged activation has deleterious effects on cardiac structure and performance, leading to the development of symptomatic HF characterized by edema [4,16,23]. It compromises the protective effects of the NP and NO-mediated pathways, as well as the ACE2-Ang (1–7) axis of RAAS [16,19,20,24,25,26,27,28]. 

In HFrEF with restricted sodium intake, classical RAAS activation is driven not only by reduced cardiac function, but also by restricted sodium intake, which may adversely affect HF progression and contribute to the increased mortality reported by some epidemiologic studies [1,5,7,11,12]. 

**Figure 1:**
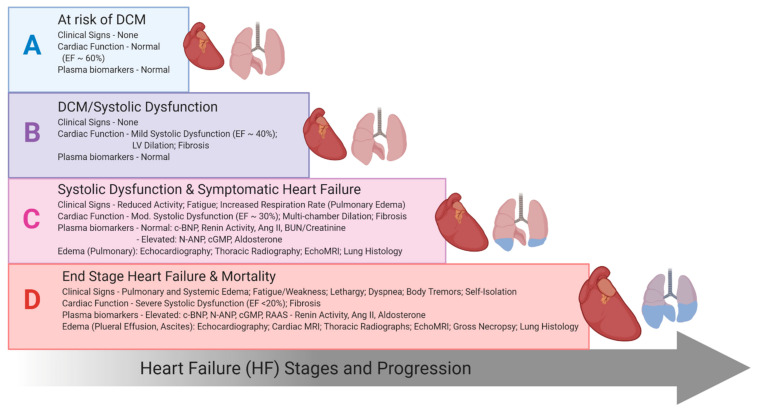
Schematic diagram of progressive heart failure stages in a normotensive dilated cardiomyopathy-heart failure with reduced ejection fraction (DCM-HFrEF) mouse model with preserved renal function based on experimental data on clinical signs, systolic function, diagnostics and blood biomarkers [16,29,31,32,33].

The goal of this study was to elucidate the longitudinal impact of a low-sodium diet (LSD, the experimental treatment) vs. a normal-sodium diet (NSD, the standard treatment) on the progressive development of HF, characterized by edema manifestation, survival and the mechanistic interplay between the classical and counter-regulatory arms of the RAAS and NP system, which modulate sodium and fluid homeostasis. These sodium diets were chosen to be translationally relevant to human diets. Definitive studies of sodium intake on HF in humans are difficult because of the multiple confounding effects of patient and ethical factors (medications, age, sex, disease severity, comorbidities including hypertension and renal dysfunction, etc.). We used a well-characterized translational model of DCM that recapitulates the progressive stages of human HF (A to D), with characteristic features of edema and biomarker profiles, in normotensive mice with preserved renal function (Figure 1) [29,30,31,32,33,34,35,36]. This model is responsive to treatments targeting RAA-NP systems with survival benefits [30,31,34,35] and complies with the AHA Scientific Statement requirements for preclinical animal models of HF [37]. This model allowed us to precisely dissect an impact of the dietary sodium on extracellular fluid retention (edema) from the impacts of preexisting pathological blood pressure and renal dysfunction. Our findings reveal that in the setting of normal blood pressure and preserved renal function, a LSD attenuates HF progression (edema, pleural effusions and mortality), through protective stimulation of the ACE2-Ang (1–7) production and NO-cGMP bioavailability, despite profound systemic classical RAAS activation.

## 2. Results

### 2.1. Dietary Sodium Restriction Suppresses Development of Pleural Effusion, Systemic Edema and Prolongs Survival 

DCM male littermate mice were randomly assigned to a LSD (treatment) or NSD (control) initiated from 28 days of age corresponding to stage A HF [32] and maintained long-term as mice progressed through A to D HF stages for ≥13 weeks as described in Methods. Littermate mice without DCM (wild type, WT) were used as a control for DCM-HF progression. 

**Figure 2:**
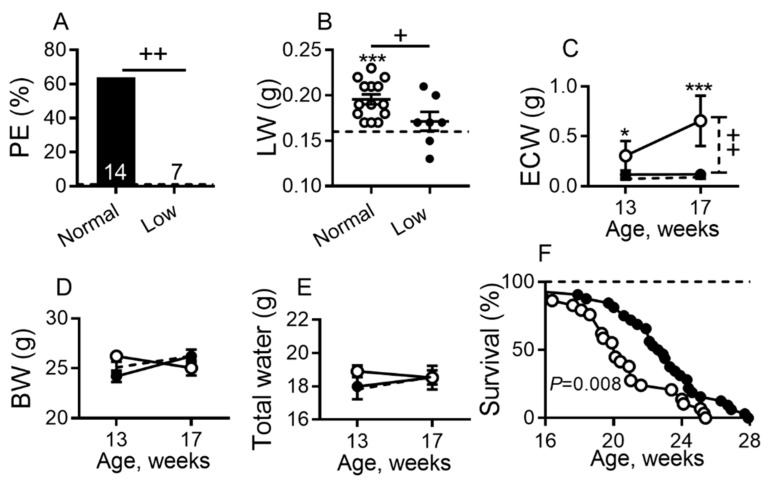
Dietary sodium restriction suppresses pleural effusions, edema and prolongs survival in mice with DCM on a normal or low sodium (low) diet. (**A**) Pleural effusions (PE) prevalence; bars represent percent of affected mice out of total mice in each group. PE was not detected in any wild-type (WT) controls. (**B**) Lung weight (LW). (**C**–**E**) Age-related changes corresponding to HF progression from C to D stages in (**C**) extracellular water (ECW), (**D**) body weights (BW), and (**E**) total water. (**A**,**B**): Groups were 20 weeks of age; DCM mice on normal-sodium diet (NSD) *n* = 14, on low-sodium diet (LSD) *n* = 7; WT mice (dashed line, *n* = 9). (**C**–**E**): DCM mice enrollment *n* = 9–11 per group; WT (dashed line, *n* = 14–15 per group). (**F**) Kaplan–Meier survival curves of DCM mice receiving LSD (*n* = 32 deaths) vs. NSD (*n* = 29 deaths); WT controls (dashed line, *n* = 8–10). DCM mice on LSD (closed symbol) or NSD (open symbol). Data are presented as mean ± SEM. *** *p* < 0.001, * *p* < 0.05 (DCM vs. WT) mice, ^++^ *p* < 0.01, ^+^ *p* < 0.05 (DCM on NSD vs. DCM on LSD). Data were assessed by a Fisher’s exact test (**A**), by one-way analysis of variance (ANOVA) with Newman–Keuls multiple comparison test (**B**), by two-way ANOVA with Bonferroni multiple comparison test (**C**–**E**) or by the Kaplan–Meier method and the log-rank (Mantel–Cox) test (**F**).

Echocardiographic examination (Video S1) of sub-groups of DCM mice at 140 days of age (Stage D HF) detected fluid motion in the chest of the pleural effusion of DCM mice on a NSD but not on a LSD. The necropsy assessment confirmed echocardiographic observations and revealed that ~60% of mice on a NSD developed pleural effusions (edema fluid accumulation in the thoracic cavity outside the lung caused by fluid movement from pulmonary edema across the visceral pleura), while no mice on a LSD had pleural effusions (*p* < 0.01; Figure 2A). Similarly, lung weights were significantly increased in mice on a NSD (*p* < 0.001) when compared with WT controls (Figure 2B) on a NSD, consistent with increased pulmonary fluid retention (pulmonary congestion, alveolar and intra-alveolar edema identified by lung histology, chest radiography and cardiac magnetic resonance imaging (MRI)) as we have reported [31,32,33,34]. Lung weights were significantly reduced in mice on a LSD vs. NSD (*p* < 0.05; Figure 2B). 

The effect of a LSD on systemic water retention was assessed by measuring total body extracellular water (ECW) or free water, using noninvasive quantitative magnetic resonance (QMR) for body composition monitoring, as we reported previously [34,35,38]. DCM mice on a NSD had significantly increased ECW when compared to WT littermate controls at 13 (Stage C HF, *p* < 0.05) and 17 (Stage D HF, *p* < 0.01) weeks of age (Figure 2C). DCM mice on a LSD showed roughly a 5.5-fold decrease vs. the DCM group on NSD (*p* < 0.001; Figure 2C). There was no difference in ECW values up to 17 weeks of age in DCM mice on a LSD vs. WT littermate controls. Body weights (Figure 2D) and total water measurements (Figure 2E) were relatively comparable among all groups. 

Consistent with its effect on decreasing the development of edema and effusions, a LSD significantly increased median survival in DCM mice by comparison to a NSD (23 vs. 20 weeks, *p* < 0.001; Figure 2F). 

### 2.2. Dietary Sodium Restriction Does Not Modulate Systolic Function 

**Figure 3:**
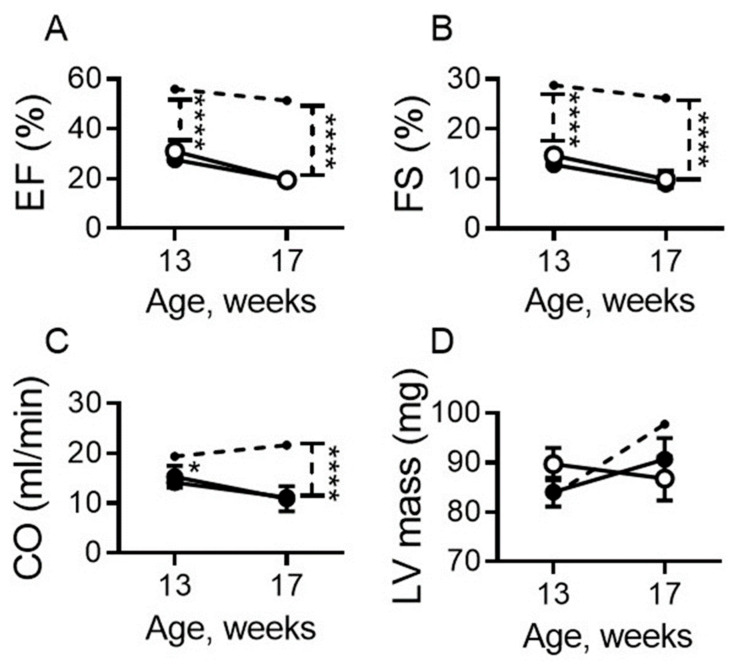
Dietary sodium restriction does not modulate cardiac systolic function in mice with DCM. (**A**–**D**) Age-related changes in left ventricular systolic function measured as ejection fraction (EF; **A**), fractional shortening (FS; **B**), cardiac output (CO; **C**), and left ventricle mass (LV mass; **D**). DCM mice on LSD (closed symbol) or NSD (open symbol) (enrollment *n* = 11–14 per group), and WT mice (dashed line, *n* = 8–10). Data are presented as mean ± standard error of the mean (SEM). **** *p* < 0.0001, * *p* < 0.05 (DCM vs. WT) as determined by two-way ANOVA with Bonferroni multiple comparison test.

Ejection fraction (EF, *p* < 0.0001; Figure 3A) and fractional shortening (FS, *p* < 0.0001; Figure 3B) were similarly decreased in DCM mice on both diets and were significantly less than WT controls (Figure 3A,B) up to 17 weeks of age. Cardiac output (CO), was also similarly decreased in DCM mice on both diets by comparison to WT controls starting at 13 weeks of age; it declined further by 17 weeks of age in DCM mice irrespective of diet (*p* < 0.0001; Figure 3C). Left ventricular mass was not statistically different in DCM mice vs. WT controls (Figure 3D) as previously reported for this model [35].

### 2.3. Dietary Sodium Restriction Stimulates Systemic Classical Renin-Angiotensin-Aldosterone System (RAAS) and Counter-Regulatory RAAS without Affecting Blood Pressure and Renal Function 

**Figure 4:**
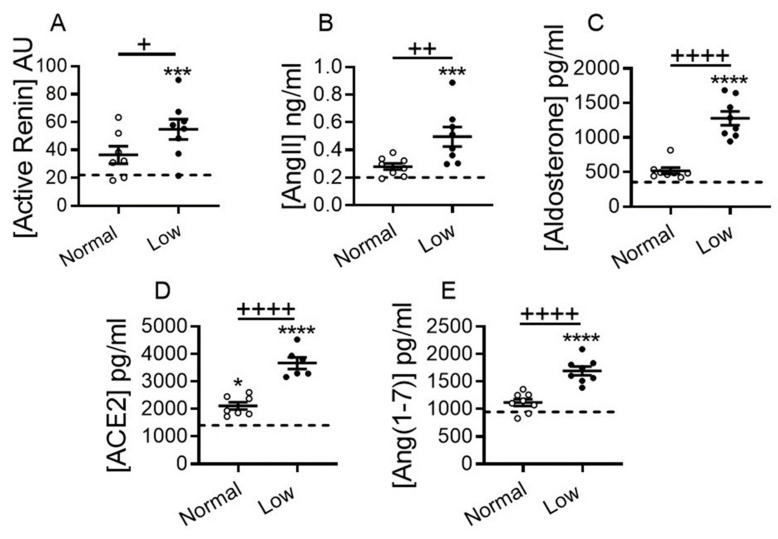
Dietary sodium restriction activates classical and non-classical renin-angiotensin-aldosterone system (RAAS) in mice with DCM. (**A**–**D**) Effect of dietary sodium restriction on plasma levels of the classical RAAS: (**A**) renin activity (AU, arbitrary units), (**B**) angiotensin II (AngII), (**C**) aldosterone, and counter-regulatory RAAS: (**D**) angiotensin converting enzyme 2 (ACE2) and (**E**) angiotensin (1–7) (Ang (1–7)). Number of DCM mice *n* = 7–8 per group. WT control mice (dashed line, *n* = 5–8) at 17 weeks of age. Data are presented as mean ± SEM. **** *p* < 0.0001, *** *p* < 0.001, * *p* < 0.001 (DCM vs. WT); ^++++^ *p* < 0.0001, ^++^ *p* < 0.01, ^+^ *p* < 0.05 (DCM on NSD vs. DCM on LSD) by one-way ANOVA with Newman-Keuls multiple comparison test.

We evaluated the impact of a LSD on classical and counter-regulatory systemic RAAS, blood pressure and renal function in DCM mice at 17 weeks of age. Plasma renin activity, angiotensin II and aldosterone levels were not significantly elevated in DCM mice vs. non-DCM WT controls on a NSD (Figure 4A–C). However, these plasma markers were significantly upregulated in DCM mice on a LSD vs. WT controls (*p* < 0.001 for both Figure 4A,B; and *p* > 0.0001; Figure 4C). Similarly, plasma renin activity (*p* < 0.05; Figure 4A), angiotensin II (*p* < 0.01; Figure 4B) and aldosterone levels (*p* < 0.0001; Figure 4C) were markedly increased in DCM mice on a LSD vs. NSD. In parallel, plasma levels of non-canonical RAAS markers ACE2 and Ang (1–7) were significantly higher on a LSD vs. NSD or by comparison to WT mice (*p* < 0.0001; Figure 4D,E).

**Figure 5:**
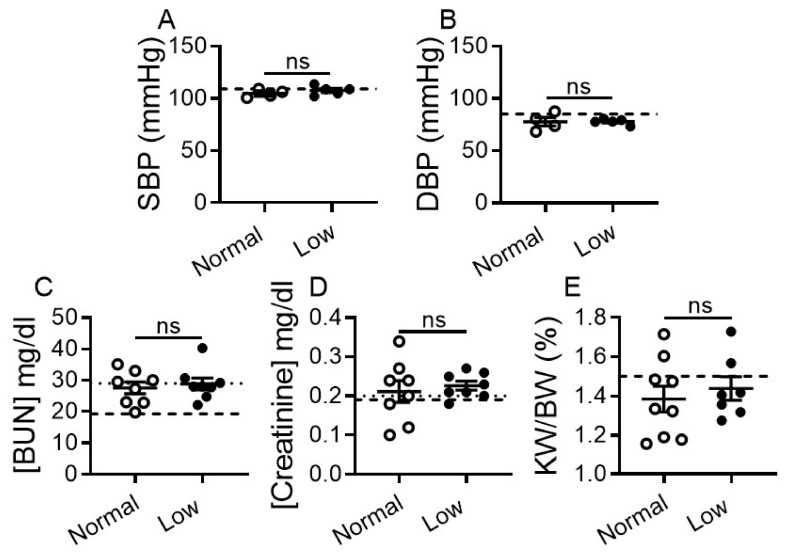
Dietary sodium restriction does not affect blood pressure and renal chemistry values in mice with DCM. (**A**) Systolic blood pressure (SBP). (**B**) Diastolic blood pressure (DBP). Three to five consecutive recordings were used and graphs were plotted as mean ± SEM. DCM mice on NSD *n* = 4, on LSD *n* = 5; WT mice (dashed line, *n* = 8). (**C,D**) Plasma levels of blood (**C**) urea nitrogen (BUN), and (**D**) creatinine. Number of DCM mice *n* = 8 per group. Dotted lines indicate upper limits of normal range of BUN and creatinine, and dashed lines represent WT control levels (*n* = 8). (**E**) Kidney weight to body weight ratio (KW/BW). Number of DCM mice *n* = 7–9 per group. WT control mice (dashed line, *n* = 9). Data are presented at 17 weeks of age, as mean ± SEM. ns = non-significant as assessed by one-way ANOVA with Newman-Keuls multiple comparison test (**A**,**B**,**E**) or by unpaired *t*-test (**C**,**D**).

Systolic and diastolic blood pressure (SBP and DBP) remained unchanged and within normal range in DCM mice regardless of diet as compared with WT mice (Figure 5A,B). 

Renal function in DCM groups was evaluated by blood urea nitrogen (BUN) and creatinine plasma levels. BUN levels were statistically higher in DCM vs. WT control mice, but remained within the normal reference range [39] in DCM mice irrespective of diet (Figure 5C). Plasma creatinine levels were unchanged in LSD and NSD groups and remained within normal reference range [39] (Figure 5D). Kidney weight to body weight ratios (KW/BW) were comparable between LSD and NSD groups and were not different vs. WT controls (Figure 5E).

### 2.4. Impact of Dietary Sodium Restriction on Systemic Levels of Natriuretic Peptide System Components and 3′-5′-Cyclic Guanosine Monophosphate (cGMP) Generation

**Figure 6:**
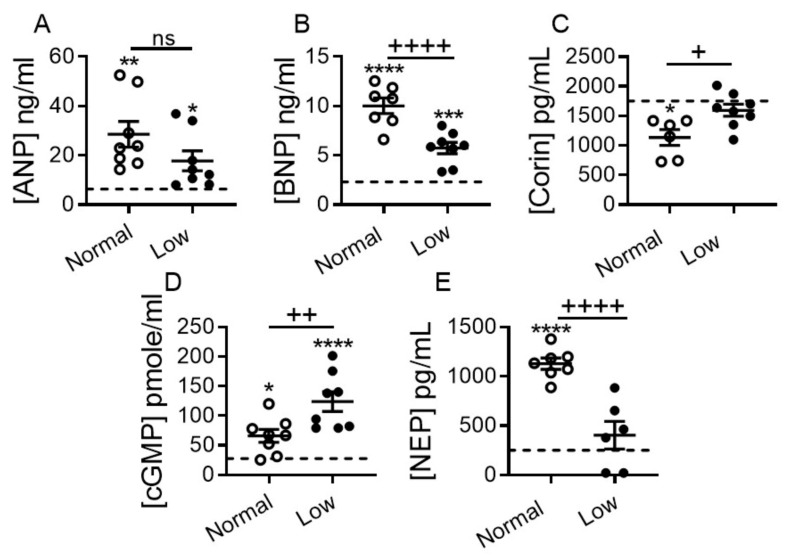
Impact of dietary sodium restriction on heart failure plasma biomarkers in mice with DCM. (**A**) Plasma levels of ANP, (**B**) BNP, (**C**) corin, (**D**) cGMP, and (**E**) neprilysin (NEP). All groups were 17 weeks of age. Number of DCM mice *n* = 6–8 per group. WT mice (dashed line, *n* = 7–8). Data are presented as mean ± SEM. **** *p* < 0.0001, *** *p* < 0.001, ** *p* < 0.01, * *p* < 0.05 (DCM vs. WT), and ^++++^ *p* < 0.0001, ^++^ *p* < 0.01, ^+^ *p* < 0.05 (DCM on NSD vs. DCM on LSD), ns = non-significant. Data were analyzed by one-way ANOVA with Newman–Keuls multiple comparison test.

The NP system promotes diuresis, natriuresis and vasodilation by counterbalancing the effect of RAAS. However, despite significant increases in circulating atrial natriuretic peptide (ANP) and B-type natriuretic peptide (BNP) levels, their actions become blunted during symptomatic HF Stages C-D [5,24,25]. We evaluated the impact of dietary sodium restriction on circulating levels of ANP and BNP, as well as corin, their activating enzyme, and neprilysin (NEP). As expected, plasma ANP (*p* < 0.01), BNP (*p* < 0.0001), cGMP (*p* < 0.05) and NEP (*p* < 0.0001) levels were significantly elevated, while corin (*p* < 0.05) levels were significantly reduced in DCM mice on a NSD vs. non-DCM WT controls on a NSD (Figure 6). BNP and NEP plasma levels were significantly lower in the LSD vs. NSD group (*p* < 0.0001 for both; Figure 6B,E); there was also a trend toward decreased ANP levels (Figure 6A). Corin (*p* < 0.05, Figure 6C) and cGMP (*p* < 0.01, Figure 6D) levels were significantly higher in the LSD group. ANP and BNP plasma levels remained significantly higher in the LSD group than WT controls (Figure 6A,B), while plasma corin and NEP levels were normalized toward WT levels (Figure 6C,E). cGMP plasma levels were increased in DCM mice irrespective of diet vs. WT controls (Figure 6D), but were two-fold higher in the LSD vs. NSD group (Figure 6D, *p* < 0.01).

### 2.5. Dietary Sodium Restriction Stimulates Systemic Ang (1–7), NO and cGMP Generation 

**Figure 7:**
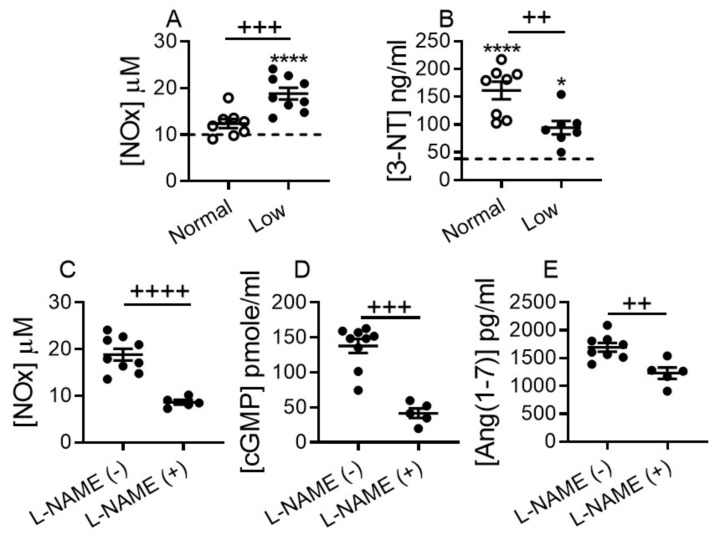
Low-sodium diet increases the nitric oxide (NO) plasma levels in mice with DCM. (**A**) Total nitrite (NO_2_^−^) and nitrate (NO_3_^−^) plasma levels (NOx = NO_2_^−^ + NO_3_^−^). (**B**) 3-nitrotyrosine levels, (3-NT). DCM mice on NSD *n* = 8, on LSD *n* = 7–9 per group. (**C**–**E**) Suppression of plasma levels of (**C**) total nitrite/nitrate, (**D**) cGMP and, (**E**) Ang (1–7) by short-term L-NAME (N-nitro-L-arginine methyl ester) tail vein infusion. Number of DCM mice L-NAME(−) *n* = 8–9 per group; L-NAME(+) *n* = 5 per group. WT control mice (dashed line, *n* = 8). Data are presented as mean ± SEM, **** *p* < 0.0001, * *p* < 0.05 (DCM vs. WT), and ^++++^ *p* < 0.0001, ^+++^ *p* < 0.001, ^++^ *p* < 0.01 (DCM on LSD vs. DCM on NSD). Data were analyzed by one-way ANOVA with Newman–Keuls multiple comparison test (**A**,**B**), by unpaired *t*-test (**C**,**E**) or by Mann–Whitney test (**D**).

Ang (1–7) mediates natriuresis and vasodilation in part through a promotion of NO release, which in turn represents an alternative mechanism that stimulates cGMP production, and attenuates systemic oxidative-nitrosative stress [20,21,40,41]. To determine if dietary sodium restriction boosts systemic NO production in DCM mice, we measured total concentration of stable NO metabolites, nitrates (NO_3_^−^) and nitrite (NO_2_^−^) in plasma. Total plasma levels of NO_2_^−^ and NO_3_^−^ were not significantly altered in DCM mice on a NSD vs. WT controls; however, levels were significantly increased in DCM mice on a LSD vs. NSD or WT groups (*p* < 0.0001; Figure 7A). Plasma levels of 3-nitrotyrosine (3-NT), a marker of oxidative-nitrosative stress, were markedly elevated above WT controls in DCM mice on a NSD, and significantly less elevated on a LSD. 3-NT (oxidative-nitrosative stress marker) levels were significantly (~2-fold) down-regulated in LSD vs. NSD group (Figure 7B).

To evaluate whether the elevated plasma cGMP and Ang (1–7) levels seen in DCM mice on a LSD were linked to increase NO production, we snap-suppressed the NO system in a sub-group of DCM mice by a single bolus injection of potent NO synthase inhibitor L-NAME at a concentration that influences cGMP production without modulating BP, as previously reported [42]. Plasma levels of total NO metabolite (*p* < 0.0001; Figure 7C), cGMP (*p* < 0.001; Figure 7D) and Ang (1–7) (*p* < 0.01; Figure 7E) were significantly suppressed in the DCM mice on LSD injected with L-NAME vs. saline, as measured 4 h post injection, and approaching corresponding levels detected in DCM mice on NSD (Figure 4E and Figure 6D). Hence, elevated plasma levels of cGMP detected in DCM mice on the LSD are likely generated through the NO- rather than NP system-related mechanism.

### 2.6. Impact of Dietary Sodium Restriction on Cardiac Expression of Nitric Oxide Synthases (NOS) and Phosphodiesterases 

**Figure 8:**
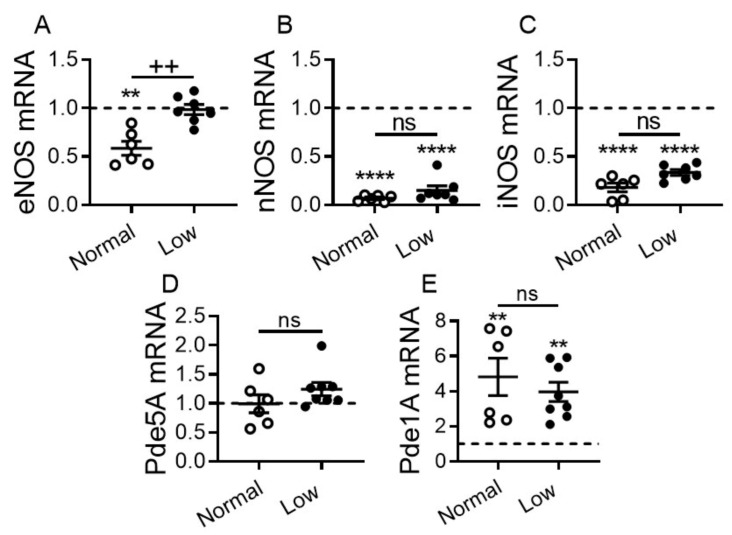
Effect of dietary sodium restriction on cardiac expression of nitric oxide synthases (NOS) and phosphodiesterases (Pde) in mice with DCM. Cardiac transcript levels of (**A**) eNOS, (**B**) nNOS, (**C**) iNOS, (**D**) Pde5A, and (**E**) Pde1A. Number of DCM mice *n* = 6–8 per group. WT control mice (dashed line, *n* = 5–6). Data are presented as mean ± SEM. **** *p* < 0.0001, ** *p* < 0.01 (DCM vs. WT), ^++^ *p* < 0.01 (LSD vs. NSD), ns = non-significant. Data were analyzed by one-way ANOVA with Newman–Keuls multiple comparison test.

The cardiac NO-cGMP pathway is known to be dysregulated in HF [43]. Hence, we measured cardiac transcript levels of all three NOS that contribute to cardiac NO-cGMP production and levels of Pde1A and Pde5A that contribute to cGMP degradation. eNOS, nNOS and iNOS levels were significantly downregulated in DCM mice on a NSD vs. WT littermates. Levels of eNOS were significantly elevated in DCM mice on a LSD vs. NSD (*p* < 0.01) and were comparable to WT controls (Figure 8A). There were non-significant trends toward increased nNOS and iNOS in DCM mice on a LSD vs. NSD, although these levels remained significantly decreased vs. WT controls irrespective of diet (*p* < 0.0001, Figure 8B,C). Pde5A levels were not statistically different in all three groups, but trended higher in DCM mice on a LSD vs. NSD (Figure 8D). Pde1A levels were increased significantly in DCM mice regardless of diet vs. WT controls (*p* < 0.01; Figure 8E). Pde1A levels trended lower in DCM mice on a LSD vs. NSD (Figure 8E). 

## 3. Discussion

Although sodium restriction has been recommended for treatment of HF, the efficacy and safety of this approach has not been established [1,5,6,7,8,12,15,44,45]. Our experimental data show that in normotensive DCM, with progressive HF [29,30,31,32,33,34,35] and preserved kidney function, LSD intake for ≥13 weeks blocked the progression of HF by preventing total body extracellular water retention, lung edema and pleural effusions. Importantly, LSD significantly prolonged survival by 15%, which is numerically analogous to increasing the life expectancy of a 75-year-old by 11 years. LSD activated the systemic classical RAAS, but did not cause pathologic changes in systolic or diastolic blood pressures or kidney function, as assessed by plasma levels of BUN and creatinine, and by kidney:body weight ratios. Sodium restriction favorably altered HF plasma biomarkers including BNP, corin, NEP and 3-NT. It increased eNOS, ACE2, Ang (1–7), NO and NO-related cGMP production, but did not significantly alter systolic function.

The NP system is an important regulator of sodium and water excretion, which counters the effects of classical RAAS through cGMP-related signaling [17]. However, as HF progresses, the potency of the NP system may diminish due in part to reduced levels of the pro-ANP convertase corin, impaired pro-ANP/pro-BNP cleavage, downregulation of renal NPs receptors and/or degradation of ANP by NEP [5,16,24,25,26,46,47,48,49]. Plasma levels of cGMP, a surrogate biomarker of clinical and experimental symptomatic HFrEF [24,30,32,33,34,35], were elevated approximately two-fold in DCM mice on a LSD, despite the reduction of ANP/BNP plasma levels. In contrast to DCM mice on a NSD, levels of corin (which cleaves and activates pro-ANP) and NEP (which degrades ANP) [48,49] were not significantly different in DCM mice on a LSD vs. WT littermate mice. This suggests that NPs may be more effective in producing cGMP in DCM on a LSD.

The Ang (1–7)–Mas/AT_2_ receptors_-_NOS-NO network are also a potent stimulator of cGMP production and regulator of cardiac function and sodium/water excretion, which counterbalances the effects of the classical RAAS [16,19,20,21,41]. In HF, the potency of Ang (1–7) and NO may be diminished, mostly because of reduced levels of ACE2, a primary enzyme for generating angiotensin and impaired availability of NO Ang (1–7) [20,28]. Ang (1–7) and NO production and activity in healthy humans and rodents are known to be sensitive to sodium status and are negatively associated with dietary sodium consumption [50,51,52]. Hence, we hypothesized that in HF Ang (1–7) and NO may be suppressed by pathological sodium retention. We found that a LSD significantly (40–50%) boosted plasma ACE2, Ang (1–7) and NO levels in DCM mice vs. NSD. The elevation of cGMP plasma levels in DCM mice on a LSD were (~3-fold) seems likely due to the NOS-NO axis rather than to NP system network, as suppression of NOS by L-NAME reduced total plasma NO levels (~2-fold) and cGMP (~3-fold) to the levels detected in DCM mice on a NSD. The elevation of Ang (1–7) plasma levels (30%) in DCM mice on a LSD were also reduced by NOS-NO suppression by L-NAME. The exact mechanism by which suppression of NOS-NO might lead to reduction of Ang (1–7) levels is not yet understood. One possibility is that the NOS-NO suppression might interdependently and negatively influence Ang (1–7) formation in tissues and/or by the endothelium. The NOS-NO suppression also might negatively modulate the process of Ang (1–7) diffusion into circulation. NOS-NO inhibition by L-NAME markedly suppressed cardiac ACE2 transcript expression and activity in C57BL/6J WT mice and in chimeric double-transgenic mice with human renin/human angiotensinogen genes [53]. However, data about the alteration of cardiac Ang (1–7) formation are not available from same report, because the cardiac Ang (1–7) levels were below detection level in all experimental groups with or without L-NAME. The authors suggested that the cardiac produced Ang (1–7) diffused into extra cellular fluid and blood stream [53].

One of the most profound effects of LSD was to increase the survival of DCM mice. Consistent with this finding, a LSD also significantly suppressed plasma levels of the nitrosative stress marker 3-NT, which is increased in patients with HF and in DCM mice on a NSD [40,54]. While we found no change in systolic function related to a LSD, we did not directly assess the potential effects of dietary sodium on cardiac tissue. Studies have suggested that the effects of sodium restriction may be even more profound in experimental models, which unlike ours, are associated with significant hypertension [15,45]. Nevertheless, our results are in agreement with previous publications, which reported that elevated circulating levels of Ang (1–7) were associated with attenuation of HF-related systemic oxidative-nitrosative stress [21,40] and that a LSD promotes NO bioavailability, improves endothelial dysfunction and reduces oxidative stress [55]. To better understand the protective contribution of the ACE-2–Ang (1–7)–NO–cGMP axis on HF progression under restricted sodium consumption in DCM, the impact of antagonists of Ang (1–7) receptors MAS and AT2 (angiotensin II receptor types) [18,21,22,56] or Ang (1–7) administration should be explored. Since the Ang (1–7) axis is compromised by SARS-CoV-2 infections [57], it is tempting to speculate that the augmentation of Ang (1–7) observed with LSD might be of benefit in COVID-19 disease.

In conclusion, a LSD markedly reduced HF progression in a translationally-relevant, normotensive model of DCM-HFrEF with preserved kidney function. The LSD suppressed the development of pleural effusions and systemic extracellular water retention and, improved survival. The LSD did not significantly affect cardiac systolic function, blood pressure and renal function. The LSD beneficially altered plasma HF biomarkers associated with poor outcomes. Although the LSD activated the classical arm of RAAS (the renin-Ang II–aldosterone axis), it also stimulated the counter-regulatory arm of RAAS (ACE2-Ang (1–7)–NO-cGMP axis), which appears to have contributed to the overall protective effects of the LSD in experimental HF.

## 4. Materials and Methods

### 4.1. Experimental Mice 

Experimental animal studies were approved by the Institutional Animal Care and Use Committees at the University of Tennessee Health Science Center (Protocol 15-050.0, approved 09 July 2015; Protocol 17-059.0, approved 26 July 2017) or the University of Arizona College of Medicine–Phoenix (Protocol 17-303, approved 11 December 2017), and were performed within AAALACi accredited facilities in accordance with National Institute of Health (NIH) Guide for the Care and Use of Laboratory Animals and with the Animal Research: Reporting In Vivo Experiments (ARRIVE) guidelines. All the experimental studies were randomized. All experimental settings, measurements and analysis were performed blindly. Mice were not excluded from the studies and no data point was omitted. Mice health, behavioral and death records were monitored and reported daily. Mice were housed under a 12:12 light–dark cycle, in the same racks of the individually ventilated caging system.

The impacts of LSD on progression of DCM and HF were examined in male littermate mice with DCM on a C57BL/6J background. DCM mice express a cardiomyocyte-specific dominant-negative CREB transcription factor, and reproducibly progress through Stages A-D of human HF, although kidney function remains within a normal range [27,28,29,30,31,32,33,34]. Mice were randomly assigned to an ad libitum maintenance of identical diets except for sodium content: a common laboratory diet for mice of 0.3% sodium (NSD, Envigo Teklad 7912; Madison, WI, USA) or low 0.05% sodium diet (LSD, custom ordered based on Envigo Teklad 7034; Madison, WI, USA). Diets were otherwise similar in other components, including potassium (7912–0.8% and 7043–0.9%). Diets were initiated from 28 days of age corresponding to stage A HF [30] and maintained until experimental end-point or natural death. A specific number of mice in each subgroup were randomly designated for survival studies or terminal end-point at 13 (stage C HF), 17 and 20 (stage D HF) weeks of age for tissue and blood collection (via cardiocentesis in prepared EDTA-aprotinin syringes to block coagulation and proteolysis) as previously reported [28,29,30,31,32,33,34]. Mice were euthanized with an overdose of inhaled 5% isoflurane (IsoFlo, Zoetis Inc., Surrey, England, UK) and death was confirmed by the absence of respiration and heartbeat.

### 4.2. Mouse Diets with Different Sodium Concentration 

A NSD (0.3% sodium or 675 mg/kg/day for 20 g mouse) is equivalent to a 55 mg/kg/day or 3300 mg daily sodium consumption for a 60 kg human i.e., human equivalent dose (HED) and is comparable to the average American sodium consumption of about 3400 mg/day and less than the average male diet of 4200 mg/day [8,35]. The mouse LSD (0.05% sodium i.e., 112.5 mg/kg/day) equals 9 mg/kg/day or a HED of 550 mg/day, which is higher than the minimum required dose for human sodium consumption. The HED was calculated by the formula: [HED (mg/kg) = Mouse dose (mg/kg) × Mouse Km (3)/Human Km (37)] as described elsewhere [58], where Km is the factor for converting mg/kg dose to mg/m^2^ dose. In a 20 g mouse Km = 3, and in a 60 kg human Km = 37.

### 4.3. Pleural Effusion and Lung Assessment 

Pleural effusion (PE) was accessed by cardiac echocardiography following by necropsy analysis of the mouse thoracic cavity as evidenced by visual examination: thoracic cavity and lungs were immersed in the pleural fluid as reported [31,33,34].

### 4.4. Extracellular Water Analysis by Quantitative Magnetic Resonance 

Systemic extracellular water (ECW) or free water was objectively recorded longitudinally as HF progresses from C (13 weeks) to D (20 weeks) stages in fully conscious and minimally restrained mice using quantitative magnetic-resonance (QMR) technology (EchoMRI 4-in-1 Analyzer, Echo Medical Systems, Houston, TX, USA) as we previously described [34,35,38]. After examination (~1.5 min recording time), the fully conscious mice were placed back into the housing boxes. Body composition, including body weight and total water were measured as the gross controls.

### 4.5. Cardiac Echocardiography 

Transthoracic echocardiograms were performed using Vevo 2100 Imaging System (FUJIFILM VisualSonics, Inc., Toronto, ON, Canada) as we have described previously [30,31,32,33,34,35,36,38,59]. Mice were anesthetized with 3% of inhaled isoflurane in medical grade oxygen continuously via nose-cone over the time of examination. Two-dimensional and M-mode images of the heart and vasculature were obtained from the parasternal long and short axis acoustic windows. Analysis was performed using Vevo LAB software (3.1.0, FUJIFILM VisualSonics, Inc., Toronto, ON, Canada) with three cardiac cycles traced to produce mean values. Heart function and morphometrics were measured directly or calculated using standard equations within the software. All measurements were recorded under identical physiological conditions: rectal body temperature 37 ± 0.5 °C and heart rate 450 ± 50 bpm.

### 4.6. Blood Pressure Measurements 

Systolic and diastolic blood pressure were measured using a non-invasive tail cuff system (Coda 6; Kent Scientific Corp., Torrington, CT, USA) as we described previously [31].

### 4.7. Renal Evaluation 

Blood urea nitrogen (BUN) and creatinine levels were measured in plasma samples by clinical laboratory tests using Vitros 250 Chemistry Analyzer (Ortho Clinical Diagnostics, Rochester, NY, USA) [29]. Mice and dissected kidneys were weighed and kidney weight (KW) to body weight (BW) ratios (%) (KW/BW) were calculated.

### 4.8. Plasma Biomarker Measurements 

Plasma levels of target proteins were measured by enzyme-linked immunosorbent assay (ELISA) as previously reported [30,31,32,33,34,35,36,59]: ANP (as N terminus-ANP), BNP (as C terminus-BNP) and angiotensin II (Phoenix Pharmaceuticals, Inc., Burlingame, CA, USA); cGMP (Enzo Life Science Inc., Farmingdale, NY, USA); corin (USCN Life Science Inc., Houston, TX, USA); Ang (1–7) (Wuhan Fine Biotech Co., Ltd., Wuhan, China); aldosterone (Abcam Inc., Cambridge, MA, USA) and neprilysin (Boster Biological Technology, Pleasanton, CA, USA). ACE2 and 3-Nitrotyrosine (3-NT) levels in plasma samples were measured using the mouse ACE2 (Abcam Inc., Cambridge, MA, USA) and OxiSelect Nitrotyrosine (Cell Biolabs, Inc., San Diego, CA, USA) ELISA kits. The proper dilution factors for target proteins were determined in the laboratory.

Renin enzymatic activity in plasma samples were measured and quantified by cleavage of exogenous fluorescence resonance transfer (FRET) peptide substrates optimized for mouse renin, FRET-QXL™520/5-FAM (AnaSpec, Fremont, CA, USA) as previously reported [16,31,33,34,35].

### 4.9. Nitric Oxide (NO) Detection 

NO levels in plasma samples were estimated by measuring the sum of stable oxidative metabolites (NOx), nitrite (NO_2_^−^) and nitrate (NO_3_^−^), which is considered to be the best index of total NO production. Nitrite/nitrate concentration in ultra-filtered (YM-10 tubes (Millipore, Burlington, MA, USA) plasma samples was measured using a commercially available assay kit (Nitrate/Nitrite Colorimetric Assay Kit; Cayman Chemical, Ann Arbor, MI, USA) utilizing the Griess assay.

### 4.10. Suppression of NO Production by N-Nitro-L-Arginine Methyl Ester (L-NAME)

To snap-suppress NO bioactivity under sodium restriction, the subgroup of DCM mice on LSD was randomly assigned for L-NAME (N-nitro-L-arginine methyl ester; Sigma, St. Louis, MO, USA), potent inhibitor of NO synthase (NOS), treatment by a single tail vein slow-infusion of 200 µL over 1 min at a concentration (1 mg/kg in saline, at 37 °C) that does not modulate blood pressure, but influences cGMP production as reported [40]. Mice were euthanized 4 h after L-NAME infusion; blood was collected for examination.

### 4.11. Quantitative Real-Time Polymerase Chain Reaction (qRT-PCR)

Quantitative real-time polymerase chain reaction (qRT-PCR) was performed as we described previously [30,31,32,35,36,59]. Total RNA was extracted from snap frozen heart tissue using the RNeasy^®^ Mini Kit (Qiagen, Venlo, The Netherlands). First strand cDNA synthesis was performed with 1 μg of total RNA (Transcriptor First Strand cDNA Synthesis Kit, Roche, Basel, Switzerland). qRT-PCR was performed using the LightCycler^®^ 480 System. Specific primers for nitric oxide synthases (NOS) were: left; 5′-ccagtgccctgcttcatc-3′, right; 5′-gcagggcaagttaggatcag-3′ for endothelial (eNOS), left; 5′-gggctgtcacggagatca-3′, right; 5′-ccatgatggtcacattctgc-3′ for inducible (iNOS), left; 5′-catcaggcaccccaagtt-3′, right; 5′-cagcagcatgttggacaca-3′ for neuronal (nNOS); for phosphodiesterase’s (Pde) were: left; 5′-ccatcattttgaccagtgctt-3′, right; 5′-agaggccactgagaatctgg-3′ for Pde5A, and left; 5′-cagcgcctgaagggaatac-3′, right; 5′-tcaacaacgttgacatcacct-3′ for Pde1A. qRT-PCR was performed at: 95 °C for 5 min, followed by 40 cycles of 95 °C (10 s), 60 °C (30 s), and 72 °C (10 s). PCR products were confirmed by melting curve analysis using the LightCycler Software 4.0 (Roche Applied Sci., Mannheim, Germany). Samples were normalized to Polr2a (DNA-directed RNA polymerase II subunit RPB1) as an internal control. Experiments were performed in triplicate and the qRT-PCR was subjected to log transformation.

### 4.12. Statistical Analysis 

Statistical analyses were performed with Prism 7.0 software (GraphPad Software, La Jolla, CA, USA). Our previous experimental studies of DCM mice showed that a sample size of seven mice in each group was sufficient to detect an expected effect size difference between the experimental groups in our primary outcome of extracellular water of 1.67 with a two-tailed alpha = 0.05 and a power of 0.8 [35,60]. Survival was analyzed by the Kaplan–Meier method and the log-rank (Mantel–Cox) test. An unpaired *t*-test or Mann–Whitney test was used to analyze difference between two groups as appropriate. One-way analysis of variance (ANOVA) with a Newman–Keuls multiple comparison test was used to analyze differences among more than two groups. Age related differences among the groups in extracellular water were analyzed by two-way ANOVA with a Bonferroni multiple comparison test. Categorical data (pleural effusions) were analyzed by Fisher’s exact test. Differences were significant if the two-tailed *p* ≤ 0.05. Data were expressed as mean ± SEM. The details of statistical methods are described in the figure legends.

## 5. Conclusions and Translational Perspectives

Dietary sodium restriction is commonly recommended to HF patients, although supporting data are modest. Concerns have been raised about how low sodium intake activates the classical RAAS to increase plasma renin activity, angiotensin II and aldosterone production as a feedback mechanism to stimulate sodium reabsorption and maintain body fluid volume. In a randomized, blinded pre-clinical trial comparing a NSD and LSD, the LSD significantly improved HF biomarkers and reduced the progression of HF, edema and death in a normotensive experimental DCM mouse model. Although the mechanisms are not yet completely known, the beneficial outcomes of the LSD included increases in ACE2, Ang (1–7), NO and cGMP, which acted to compensate for the deleterious effects of systemic classical RAAS activation observed with this diet. While further studies are required to better define the dose-related effects of sodium restriction in humans with established HF medications, these pre-clinical data provide support for the value of a sodium-restricted diet in HF.

## 6. Patents

UA Tech Launch along with the authors have filed the patent related to the extracellular water analysis as a measure of edema.

## Data Availability

The data underlying this article will be shared on reasonable request to the corresponding author.

## Supplementary Materials

The following are available online at https://www.mdpi.com/article/10.3390/ijms22084035/s1, Video S1: Echocardiograph visualization of pleural edema.
